# Clinical characteristics and outcome of critically ill COVID-19 patients with acute kidney injury: a single centre cohort study

**DOI:** 10.1186/s12882-021-02296-z

**Published:** 2021-03-15

**Authors:** R. Lowe, M. Ferrari, M. Nasim-Mohi, A. Jackson, R. Beecham, K. Veighey, R. Cusack, D. Richardson, MPW Grocott, DZH Levett, A. Dushianthan, On behalf of the University Hospital Southampton Critical Care Team and the REACT COVID investigators, Sanjay Gupta, Julian Nixon, Michael P. W. Grocott, Denny Z. H. Levett, Michael Stewart, Ahilanadan Dushianthan, David Sparkes, Robert Chambers, Kathleen Nolan, Suzie Tanser, Jonathan Fennell, Max Jonas, Michael Celinski, Dominic Richardson, Rebecca Cusack, Benjamin Skinner, Timothy Nicholson-Robert, Mai Wakatsuki, Carin Dear, Ben Thomas, Francois Wessels, Tom Wilkinson, Anna Freeman, Hannah Burke, Ahilanadan Dushianthan, Michael Celinski, James Batchelor, Saul Faust, Gareth Thomas, Christopher Kipps

**Affiliations:** 1 General Intensive Care Unit, University Hospital Southampton NHS Foundation Trust, Tremona Road, Southampton, SO16 6YD England; 2 Renal Medicine, University Hospital Southampton NHS Foundation Trust, Tremona Road, Southampton, SO16 6YD England; 3 NIHR Southampton Clinical Research Facility and NIHR Southampton Biomedical Research Centre, University Hospital Southampton / University of Southampton, Tremona Road, Southampton, SO16 6YD England; 4 Faculty of Medicine, University of Southampton, University Hospital Southampton, Tremona Road, Southampton, SO16 6YD England

**Keywords:** COVID-19, Intensive care, Acute kidney injury

## Abstract

**Background:**

Acute kidney injury (AKI) is a common manifestation among patients critically ill with SARS-CoV-2 infection (Coronavirus 2019) and is associated with significant morbidity and mortality. The pathophysiology of renal failure in this context is not fully understood, but likely to be multifactorial. The intensive care unit outcomes of patients following COVID-19 acute critical illness with associated AKI have not been fully explored. We conducted a cohort study to investigate the risk factors for acute kidney injury in patients admitted to and intensive care unit with COVID-19, its incidence and associated outcomes.

**Methods:**

We reviewed the medical records of all patients admitted to our adult intensive care unit suffering from SARS-CoV-2 infection from 14th March 2020 until 12th May 2020. Acute kidney injury was defined using the Kidney Disease Improving Global Outcome (KDIGO) criteria. The outcome analysis was assessed up to date as 3rd of September 2020.

**Results:**

A total of 81 patients admitted during this period. All patients had acute hypoxic respiratory failure and needed either noninvasive or invasive mechanical ventilatory support. Thirty-six patients (44%) had evidence of AKI (Stage I-33%, Stage II-22%, Renal Replacement Therapy (RRT)-44%). All patients with AKI stage III had RRT. Age, diabetes mellitus, immunosuppression, lymphopenia, high D-Dimer levels, increased APACHE II and SOFA scores, invasive mechanical ventilation and use of inotropic or vasopressor support were significantly associated with AKI. The peak AKI was at day 4 and mean duration of RRT was 12.5 days. The mortality was 25% for the AKI group compared to 6.7% in those without AKI. Among those received RRT and survived their illness, the renal function recovery is complete and back to baseline in all patients.

**Conclusion:**

Acute kidney injury and renal replacement therapy is common in critically ill patients presenting with COVID-19. It is associated with increased severity of illness on admission to ICU, increased mortality and prolonged ICU and hospital length of stay. Recovery of renal function was complete in all survived patients.

**Supplementary Information:**

The online version contains supplementary material available at 10.1186/s12882-021-02296-z.

## Background

SARS-CoV-2 viral infection leading to Coronavirus 2019 (COVID-19) was declared as an emerging pandemic by the World Health Organization (WHO) in March 2020 [1]. The first case was reported in the United Kingdom (UK) on the 31st January and patient numbers rose rapidly with a corresponding increase in admissions to hospital and intensive care units (ICU) over the subsequent months. Although the majority of infected patients developed mild or no respiratory symptoms, a proportion progressed to severe lung disease characterised by acute hypoxic respiratory failure (AHRF) necessitating respiratory support or mechanical ventilation. Acute kidney injury (AKI) is relatively common in both hospitalised and critically ill COVID-19 patients [2, 3]. In a multicentre study of more than 3000 COVID-19 ICU patients from the United States America, around 20% developed acute kidney injury (AKI) requiring renal replacement therapy [3]. Similarly, in the UK, during the first wave of the pandemic a quarter of intensive care unit COVID-19 patients needed renal replacement therapy [4]. Furthermore, AKI may be associated with an ongoing requirement for renal support and prolonged hospitalization thereby imposing a significant health and resource burden.

The pathophysiology of AKI in COVID-19 is poorly understood but may involve a combination of pre-renal and intrinsic renal insults. Studies have described the virus’ affiliation for the angiotensin converting enzyme-2 (ACE-2) receptor, which is expressed in abundance in the kidney [5, 6]. The virus also directly infects tubular epithelial cells and podocytes causing significant structural damage [7]. Proteinuria and haematuria have been commonly documented and may be indicative of intrinsic renal injury [2, 8–10]. Post-mortem findings of COVID-19 patients suggest micro-vascular occlusion, endothelial injury, diffuse acute proximal tubular injury and evidence of direct damage as a consequence of SARS CoV-2 infection [7].

The majority of critically ill patients with COVID-19 require ventilatory support, but the clinical manifestations of COVID-19 vary and the characteristics and outcomes of patients with AKI have not been fully defined. Consequently we aimed to characterise the risk factors for AKI in intensive care patients with Covid-19, its incidence and patient outcomes in a single centre cohort study.

## Methods

This study was conducted in a General Intensive Care Unit (GICU) at the University Teaching Hospital in Southampton (UHS), UK. All adult patients (> 18 years old) admitted between 14/03/2020 and 12/05/2020 to the Intensive Care Unit with a diagnosis of COVID-19 confirmed via a reverse-transcriptase-polymerase-chain-reaction (RT-PCR) test were included in this study. The respiratory samples were taken from nasal and throat swabs or endotracheal tube aspirates for confirmatory analysis. Ethical approval was obtained as part of the REACT COVID observational study (A longitudinal Cohort Study to facilitate Better understanding and Management of SARS-CoV-2 infection from hospital admission to discharge across all levels of care): REC Reference 10/NW/0632 SRB Reference Number; SRB0025. Due to the nature of the study, the need for individual informed consent was waived.

Data was collected from an electronic clinical record system (*Meta-vision, iMDsoft*). This included baseline demographics, past medical history, pre-hospital medication, timings of disease onset, laboratory test results, ventilator strategies and clinical information regarding renal supportive measures and outcomes. Comorbidities included; diabetes mellitus, ischæmic heart disease, hypertension, chronic kidney disease, immunosuppression and congestive cardiac failure. We further quantified the severity of comorbidities using the Charlson’s comorbidity index [11]. Disease severity on admission to the ICU was assessed by the Acute Physiology and Chronic Health Evaluation II (APACHE II) score, Sequential Organ Failure Assessment (SOFA) score and degree of hypoxia from the ratio of arterial oxygen partial pressure (PaO_2_ in kPa and mmHg) to fractional inspired oxygen (PaO_2_/FiO_2_ ratio or P/F ratio).

Acute kidney injury was defined based by the Kidney Disease Improving Global Outcome (KDIGO) classification which depends on the serum creatinine profile during the hospital admission [12]. Briefly, AKI was detected when there is an increase in serum creatinine (SCr) by ≥26.5 μmol/L within 48 h or increase in SCr to > 1.5 times baseline occurred within the prior 7 days. Staging was classified as stage I when 1.5–1.9 times, stage II 2.0–2.9 times (or >26.5 μmol/l increase) and stage III 3 times increment (or >353.6 μmol/l increase or initiation of renal replacement therapy) from the baseline serum creatinine. As baseline we used either an estimated SCr or the lowest SCr in the first week of admission. If creatinine was found to be elevated at presentation, we reviewed the case notes for historical results within 1 year to determine if this was an acute process. If there were no historical blood results, then an estimated baseline creatinine was used to grade the AKI according to their gender, ethnicity and age (according to KDIGO criteria) [12]. Renal function recovery is defined as improvement in creatinine below 1.5 times the baseline or estimated creatinine. All admitted patients were divided into two groups; patients with AKI and without AKI. The use of vasopressors and diuretics with additional information of cumulative daily fluid balance was also documented.

Descriptive statistics were used to characterize the clinical variables of patients in the AKI and no AKI groups. The Kolmogorov-Smirnov was used to evaluate normality in continuous data. Direct comparisons between the AKI and no AKI groups were performed with the Mann-Whitney U (continuous variables) and Fisher’s exact tests (categorical variables).

## Results

Between 14th of March and 12th of May 2020, a total of 81 critically ill COVID-19 patients were admitted to the Intensive Care Unit. The median age was 57 (IQR 18) and 62% were male. The median duration of symptoms prior to hospitalisation was 7 days (IQR 5). 56.8% were white and 48.1% had a raised BMI of >30 kg/m^2^. For those with baseline creatinine available, chronic kidney disease was evident in 5 patients (8.3%) and one patient was receiving long-term dialysis for end stage renal failure secondary to diabetic nephropathy. The comorbidities recorded were diabetes mellitus (25.9%), hypertension (37%) and immunosuppression (9.9%). Of patients with immunosuppression; 3 patients had Human Immunodeficiency Virus infection (HIV), 2 had hematological malignancies, 2 were on immunosuppressive medications for autoimmune diseases and one undergoing chemoradiotherapy for intracerebral solid tumor. Twenty patients (24.7%) were documented to be on prescription medication of either an angiotensin converting enzyme II (ACE II) inhibitor or an angiotensin receptor blocker (ARB).

**Fig. 1:**
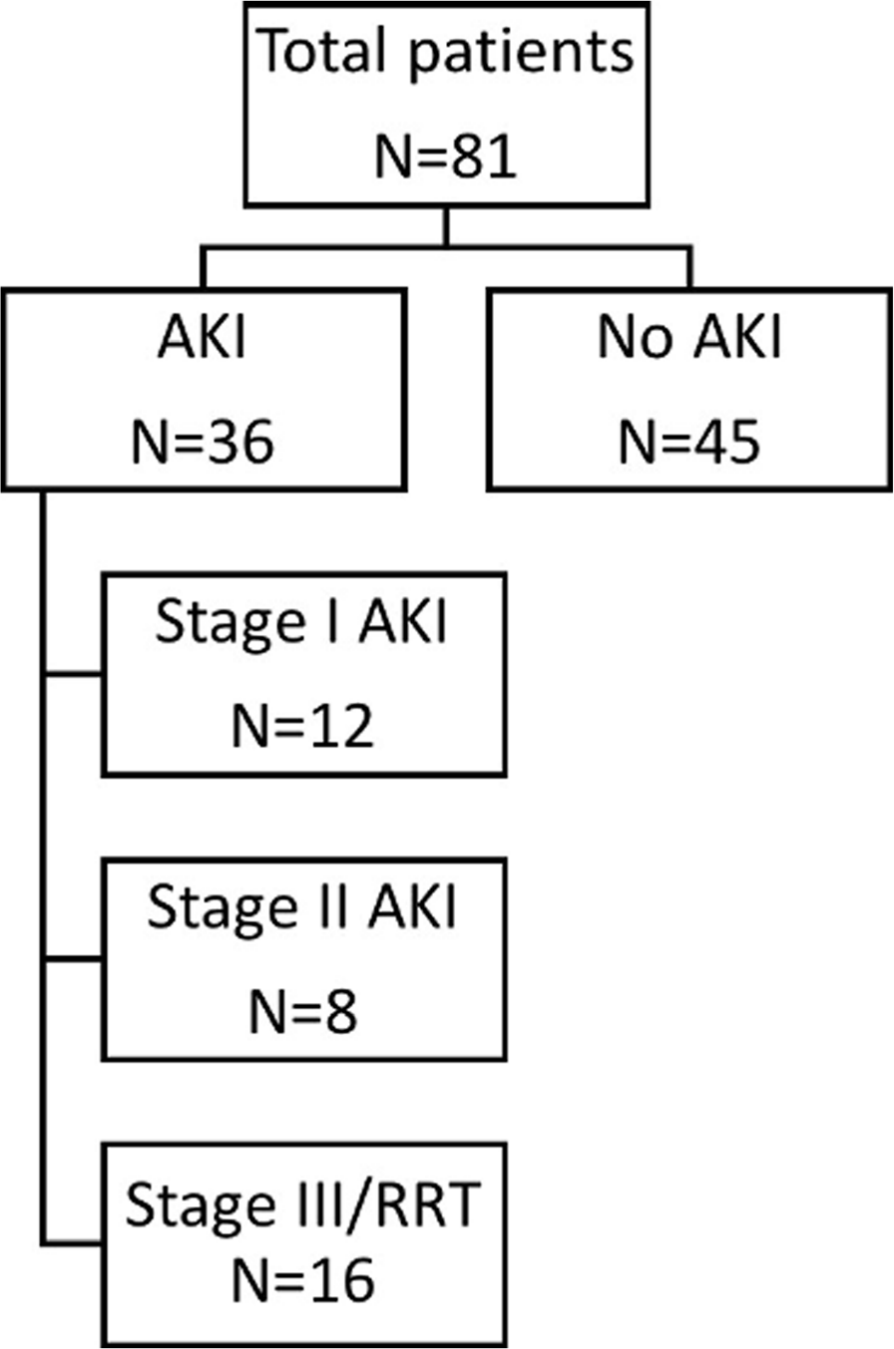
COVID-19 patient flow diagram. AKI, Acute Kidney Injury; RRT, Renal Replacement Therapy. The Acute Kidney Injury is defied according to the Kidney Disease Improving Global Outcomes (KDIGO) criteria

Thirty-six patients (44.4%) had an AKI at any time during their ICU stay. Of these, 12 (33.3%) had AKI stage I, 8 (22.2%) had AKI stage II and 16 patients (44.5%) had AKI stage III and received renal replacement therapy (RRT) (Fig. 1).

The patients with AKI were older (61 vs 50, *p* = 0.0071) with increased comorbidities as quantified by Carlson’s comorbidity index (2.5 vs 1, *p* = 0.0016) and had a higher incidence of diabetes mellitus (38.9% vs 15.6%, *p* = 0.0225) and immunosuppression (19.4% vs 2.2%, *p* = 0.0193) (Table 1). Patients with AKI had greater illness severity as determined by APACHE II (20 vs 12, *p* < 0.0001) and SOFA (5 vs 3, *p* = 0.0001) scores. The degree of acute hypoxic respiratory failure was similar in the two groups: PaO_2_/FiO_2_ ratio (P/F ratio) of 14.4 vs 15.4 kPa or 108 vs 115 mmHg, *p* = 0.0811). However, AKI patients were more likely to have received invasive mechanical ventilation (86.1% vs 37.8%, *p* = 0.0001) and vasopressor or inotropic support (91.7% vs 35.6%, p = 0.0001). The use of diuretics and corticosteroids were also more common in patients with AKI. The clinical characteristics of all patients are presented in Table 1.

**Table 1:** Patient demographics on admission, disease severity indices, intensive care interventions and admission laboratory markers from all COVID-19 admitted patients

Demographics	All patients (***N*** = 81)	AKI (***N*** = 36)	No AKI (***N*** = 45)	***P***-value
Age, years	57 (18)	61 (14)	50 (16)	*P* = 0.0071^*^
Male (%)	62%	66.7%	57.8%	*P* = 0.4930
Symptomatic days prior to hospitalisation	7 (5)	7 (7)	8 (4)	*P* = 0.1183
BMI >30 kg/m^2^, n (%)	29 (48.1%)	21 (58.3%)	18 (40%)	*P* = 0.1208
Charlson’s comorbidity index	2 (2)	2.5 (3)	1(3)	*P* = 0.0016^*^
Race/ethnic group				
White	46 (56.8%)	24 (66.7%)	22 (48.9%)	*P* = 0.1209
Black	7 (8.6%)	4 (11.1%)	3 (6.7%)	*P* = 0.6941
Asian/Indian	20 (24.7%)	5 (13.9%)	15 (33.3%)	*P* = 0.0684
Other/unknown	8 (9.9%)	3 (8.3%)	5 (11.1%)	*P* = 0.7274
**Comorbidities, n (%)**				
Chronic kidney disease	5 (6.2%)	3 (8.3%)	2 (4.4%)	*P* = 0.6511
Congestive cardiac failure	4 (4.9%)	2 (5.6%)	2 (4.4%)	*P* = 1.0000
Diabetes mellitus	21 (25.9%)	14 (38.9%)	7 (15.6%)	*P* = 0.0225^*^
Hypertension	30 (37%)	15 (41.7%)	15 (33.3%)	*P* = 0.4924
Ischemic heart disease	7 (8.6%)	2 (5.6%)	5 (11.1%)	*P* = 0.4537
Immunosuppression	8 (9.9%)	7 (19.4%)	1 (2.2%)	*P* = 0.0193^*^
Use of ACEi or ARB	20 (24.7%)	10 (27.8%)	10 (22.2%)	*P* = 0.6111
**Severity indices**				
APACHE II Score	14 (12)	20 (11.2)	12 (6)	*P* < 0.0001^*^
SOFA Score	4 (3)	5 (3)	3 (1)	*P* = 0.0001^*^
PaO_2_/FiO_2_ ratio				*P* = 0.0811
kPa	15 (5)	14.4 (4.5)	15.4 (4.8)	
mmHg	112.5 (37.5)	108 (33.7)	115.5 (36)	
**ICU interventions**				
Mechanical ventilation, n (%)	48 (59.3%)	31 (86.1%)	17 (37.8%)	*P* = 0.0001^*^
Non-invasive ventilation, n (%)	33 (40.7%)	5 (13.9%)	27 (60%)	*P* = 0.0001^*^
Vasopressor use, n (%)	49 (60.5%)	33 (91.7%)	16 (35.6%)	*P* = 0.0001^*^
Vasopressor use > 1 type, n (%)	15 (18.5%)	13 (36.1%)	2 (4.4%)	*P* = 0.0003^*^
Diuretics use, n (%)	49 (60.5%)	27 (75.0%)	22 (48.9%)	*P* = 0.0225^*^
Diuretics use > 1 type, n (%)	20 (24.7%)	14 (38.9%)	6 (13.3%)	*P* = 0.0090^*^
Corticosteroids	23 (28.4%)	15 (41.7%)	8 (17.8%)	*P* = 0.0255^*^
Pulmonary vasodilators, n (%)	11 (13.6%)	8 (22.2%)	3 (6.67%)	*P* = 0.0544
Antibiotics (any), n (%)	81 (100%)	36 (100%)	45 (100%)	*P* = 1.0000
Antivirals (any), n (%)	25 (31.9%)	12 (33.3%)	13 (28.9%)	*P* = 0.8093
**Admission laboratory profile**				
Bilirubin (mmol/l)	11 (6)	11 (9)	11 (5)	*P* = 0.8939
Creatinine (mmol/l)	72 (44)	98 (84)	65 (28)	*P* = 0.0222^*^
Creatinine kinase (U/l)	160 (240)	153 (411)	174 (237)	*P* = 0.8105
C-Reactive Protein (mg/l)	153 (106)	166 (135)	125 (82)	*P* = 0.0598
D-Dimer (mg/l)	527 (742)	942 (2157)	444 (395)	*P* = 0.0318^*^
Ferritin (mg/l)	965 (1430)	842 (963)	989 (1601)	*P* = 0.9087
HbA1c (mmol/mol)	46 (9)	48 (10)	44.5 (8)	*P* = 0.0615
LDH (U/l)	909 (572)	1026 (721)	909 (550)	*P* = 0.9745
Lymphocytes 10^9^/l	0.9 (0.7)	0.700 (0.7)	1.000 (0.6)	*P* = 0.0133^*^
Neutrophil/lymphocyte ratio	7.8 (6.8)	8.7 (6.2)	6.7 (5.4)	*P* = 0.1010
Procalcitonin (ng/ml)	0.3 (0.6)	0.6 (0.9)	0.3 (0.5)	*P* = 0.1076
HS Troponin (ng/l)	13 (26.5)	27.5 (117)	9 (8)	*P* = 0.0001^*^
White cell counts 10^9^/l	8.2 (6.5)	8.9 (7)	7.6 (5.7)	*P* = 0.2935

Data are presented as median (Interquartile Range) or numbers (percentage) unless otherwise stated. *P < 0.05 as assessed by Fisher’s Exact test for categorical variables and Mann-Whitney U test for continuous variables. *ACEi* Angiotensin Converting Enzyme inhibitor, *APACHE II* Acute Physiology and Chronic Health Evaluation II score, *ARB* Angiotensin Receptor Blocker, *BMI* Body Mass Index, *LDH* Lactate dehydrogenase, *HS Troponin* High Sensitivity Troponin, *PaO*_*2*_*/FiO*_*2*_ Ratio of arterial oxygen partial pressure to fractional inspired oxygen, *SOFA* Sequential Organ Failure Assessment score

**Fig. 2:**
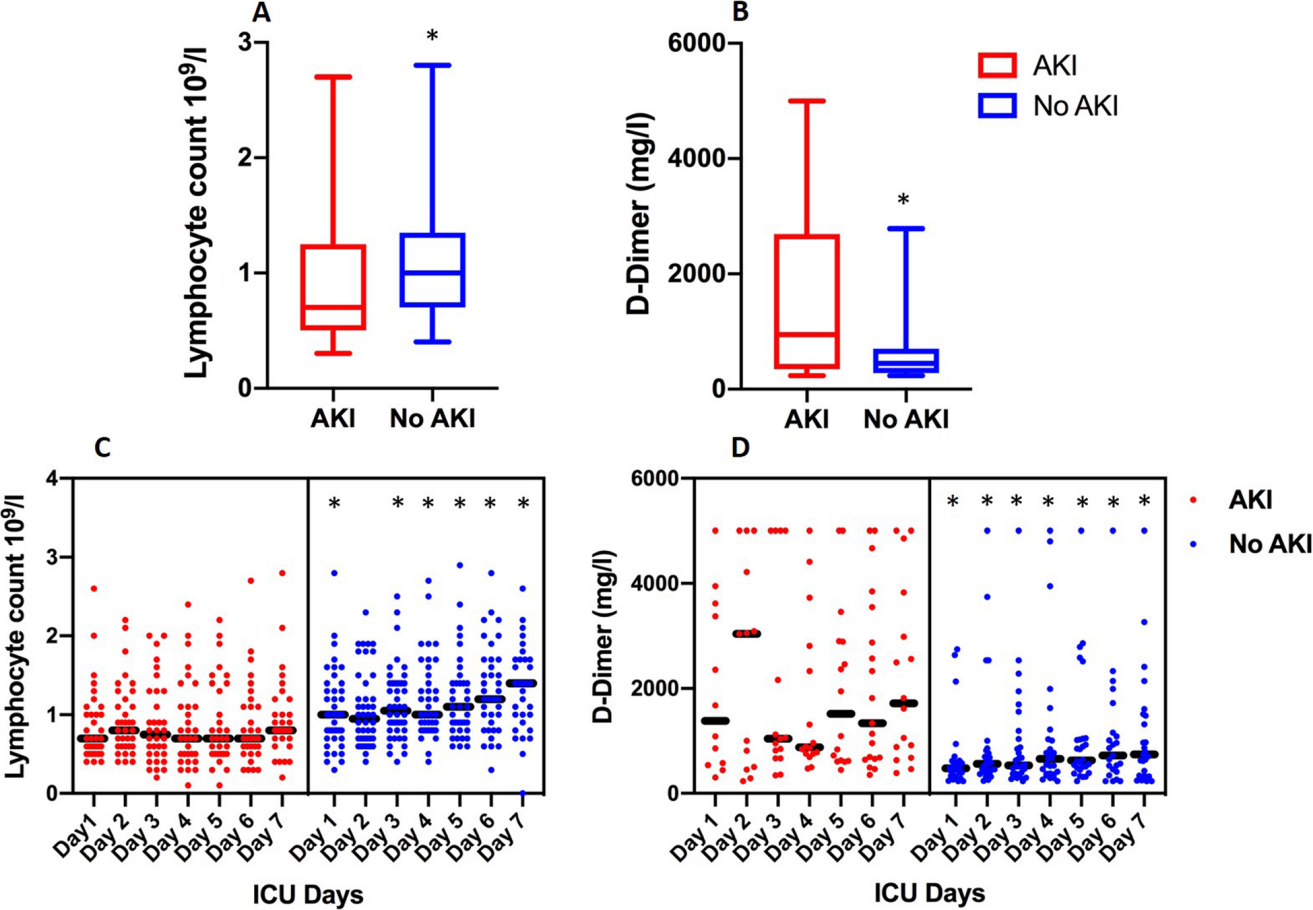
The lymphocyte counts (10^9^/l) and D-Dimer levels for Day 1 (**a**: lymphocyte, **b**: **d**-Dimer) and over the 7 days of Intensive Care Unit admission (**c**: lymphocyte counts, **d**; D-Dimer) for both groups with and without acute kidney injury. The 2A and 2B are presented as Box and Whiskers plots with median and maximum and minimum values. 2C and 2D shows scatter plots and the lines represent median values. ^*^The comparison between AKI vs No AKI group for that particular time point by Mann-Whitney U test and *P* < 0.05

On admission, patients with AKI had lower lymphocyte counts and higher D-Dimer, troponin and creatinine levels (Table 1). The lymphopenia with raised levels of D-Dimers persisted even at day 7 and in patients without AKI, whereas lymphocyte count in those without AKI continue to increase after the ICU admission (Fig. 2) and Additional file 1.

**Fig. 3:**
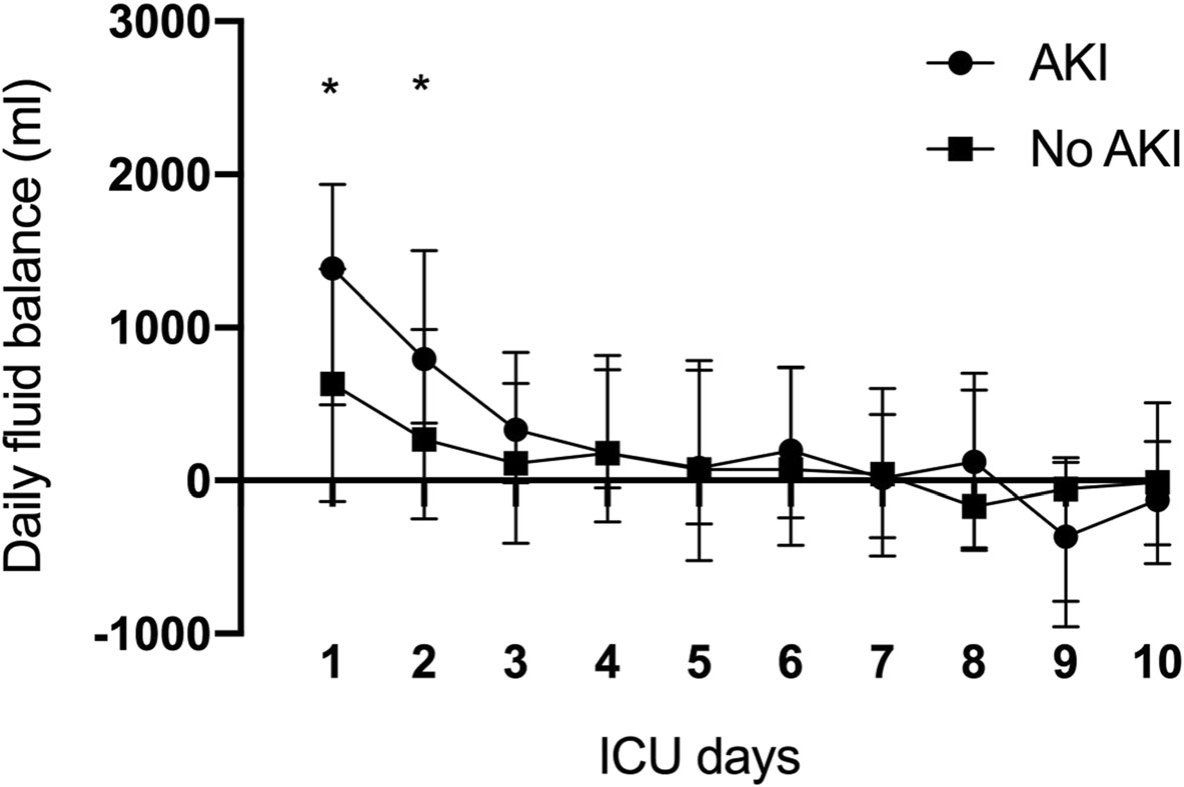
Daily cumulative fluid balance for patients with and without AKI for the first 10 days of ICU admission. Data is presented as median and interquatile ranges. ^*^The comparison between AKI vs No AKI group for that particular time point by Mann-Whitney U test and *P* < 0.05

The daily fluid balance analysis showed more positive fluid balance in the AKI group at day 1 (+670mls, 95% CI: + 1165 to + 143, *P* = 0.01) and day 2 (+497mls, 95% CI: + 880 to + 28, *P* = 0.04) (Fig. 3). For patients admitted to ICU with AKI, the median time from (we calculated from ICU admission) to documented peak AKI was 4 days (IQR 6 days) and the median peak AKI from symptom onset was 10.5 days (IQR 7.3 days). For patients who received RRT, the median time to commencing RRT was 5 days (IQR 6 days) after admission to the intensive care unit and the median duration of RRT was 12.5 days (IQR 15.5 days). The mode of renal support was primarily continuous veno-venous hemodiafiltration (CVVHDF) with citrate anticoagulation. Out of the 16 patients who received RRT, 10 received additional formal therapeutic anticoagulation with intravenous heparin. A standard augmented anticoagulation protocol was utilised for all COVID-19 patients including for those with an AKI (see Additional file 2).

**Fig. 4:**
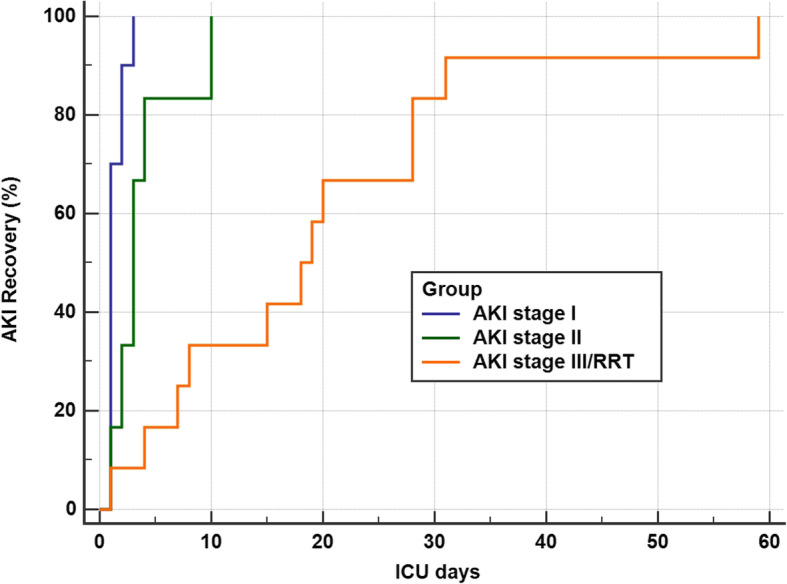
The recovery of acute kidney injury across all stages of AKI as defined by the Kidney Disease Improving Global Outcomes (KDIGO) criteria over time during the intensive care unit stay. The RRT group recovery is defined as normalisation of renal function off renal replacement therapy. AKI, Acute kidney injury; ICU, Intensive Care Unit; RRT, Renal Replacement Therapy

Outcomes are up to date as of 3st of September 2020. Among those who developed AKI and survived their acute COVID-19 illness, all patients (27/27) have fully recovered to their baseline renal function (Fig. 4). The overall hospital mortality for the patients admitted to ICU with AKI was 25% (9/36), compared to 6.7%, (3/45) in those without AKI. One patient remains hospitalised for rehabilitation (Table 2). Overall, twelve patients died. For those patients with completed outcomes, the median length of ICU and hospital stay was significantly longer in patients with AKI at 28 (IQR 34) and 39.5 days (IQR 36.5) compared to 5.5 days (IQR 11) and 15.5 days (IQR 14 days) for without AKI, respectively.

**Table 2:** Outcome of all patients with and without acute kidney injury

Outcomes	Patients with AKI (***N*** = 36)	Patients without AKI (***N*** = 45)
Length of RRT	12.5 (10.8)	N/A
Length of ICU Stay	28 (34)	5.5 (11)
Length of Hospital Stay	27 (23)	15.5 (14)
Death	9 (25.0%)	3 (6.7%)
Still Hospitalised	1 (2.8%)	0 (0%)
Still in ICU	0 (0%)	0 (0%)
Still on RRT	1^a^	N/A
Discharged Home	19 (72.2%)	42 (93.3%)

*AKI* Acute Kidney Injury, *ICU* Intensive Care Unit, *RRT* Renal Replacement Therapy. Data are presented as median (Interquartile Range) or numbers (percentage) unless otherwise stated. ^a^One patient had CKD with established long-term dialysis programme

## Discussion

In this study, we report that 45% of COVID-19 patients admitted to intensive care for respiratory support developed an AKI and 20% of the total ICU cohort required renal replacement therapy acutely for an average duration of 12.5 days. Overall hospital mortality for critically ill ICU patients with COVID-19 was 15%, compared to 25% in patients with AKI. All survived patients with AKI stage I and II and stage III/RRT had complete recovery to their baseline renal function. Risk factors for the development of AKI were older age, preexisting diabetes mellitus and immunosuppression for any reason including HIV, myelosuppression due to hematological malignancies and immunosuppressive therapy. AKI was associated with increased disease severity on admission (APACHE II and SOFA scores), mechanical ventilation, persistently raised D-Dimer and more severe lymphopenia.

AKI is an independent risk factor for increased mortality in all critical illness [13, 14]. The reported incidence of AKI among critically ill COVID 19 patients in earlier cohorts from China was approximately 20–30% and it is regarded as a marker of disease severity [15, 16]. Although the incidence of AKI in our cohort is higher than these early reports, it is comparable to the other international studies from Brazil [17] and USA [3]. Our renal replacement rate is also comparable to the reports from larger cohorts with a rate of 20–31% [3, 4]. However, in studies of mostly invasively ventilated critically ill COVID 19 patients, the incidence of AKI was reported to be as high as 75% with an RRT rate of 17.7–51% [10, 18, 19]. In contrast, our cohort was inclusive of patients with noninvasive ventilation, which may account for the difference in AKI prevalence, RRT rate and mortality.

Our finding of increased mortality in patients with AKI (25% vs 6.7%) is in keeping with other COVID-19 case cohorts [8, 9, 20, 21]. Patients with AKI had more severe illness generally, required invasive mechanical ventilation, had higher illness severity scores, persistent lymphopenia and vasopressor support, suggesting that AKI is a marker of disease severity. Immunosuppression for any reason was associated with increased incidence of AKI. It is possible that patients who are immunocompromised may have an increased severity of COVID-19 critical illness predisposing them to develop AKI [22].

The cumulative daily fluid balance suggests more positive fluid balance in the AKI group for the first 48 h of ICU admission. This in combination with the increased frequency of vasopressor and corticosteroids usage suggests that patients with AKI were sicker than patients without AKI. Among our all AKI cases, the mortality for the AKI groups, Stage I, Stage II and the RRT group are 25, 25 and 27% respectively. This is lower than the 56% national mortality in patients receiving RRT reported in the UK ICNARC outcome dataset and from other international studies [3, 4, 18]. Patients in our RRT cohort had similar age and acute severity indices (APACHE II of 21, PaO_2_/FiO_2_ 15.1 kPa or 113 mmHg) with higher mechanical ventilation rate (100%) and 92% had vasoactive agents as advanced cardiovascular support suggesting this group is comparable to the ICNARC dataset [4]. While we are unable to postulate the exact reasoning behind this better survival rate, it is likely to be multifactorial and is reflection of variations in patient’s population, resource availability and ICU interventions.

Although the exact pathophysiological mechanism of AKI in COVID-19 remains elusive, it appears to be multifactorial. Direct viral infection [7, 23], overt immune response leading to tubuloepithelial injury [24] and microvascular endothelial injury due to microthrombi formation [25, 26] are some of the underlying pathophysiological mechanisms postulated. The contribution of microthrombi formation in AKI has been found in postmortem findings, where there is erythrocyte stagnation with clot formation in the glomerular and peri-tubular capillaries in COVID-19 patients [6]. Moreover, one study demonstrated that elevated D-Dimer level and complete failure of lysis at 30 min on a thromboelastogram are predictive of significant increase in the incidence of thromboembolism and a need for haemodialysis in critically ill COVID-19 patients [26]. In our report, the development of AKI was associated with raised admission D-dimer levels and these abnormalities persisted in patients with AKI even after a week of ICU admission. This supports the fore-mentioned studies and highlights this maybe a potential contributor to the underlying pathophysiology of AKI in COVID-19 cases.

Interestingly, the use of diuretics was much more common in patients with AKI. Diuretics are often used in the intensive care setting to enhance a judicious fluid balance to improve oxygenation in patients with acute severe hypoxic respiratory failure. It is not clear whether the increased usage of diuretics contributed to the development of AKI or whether they were used to facilitate urine output and consequently improve fluid balance when AKI was already established. Similarly, AKI patients were more likely to be treated with corticosteroids. The use of corticosteroids probably reflects a more severe disease process and it is not possible to make any direct conclusions regarding cause and effect from this study.

Our study has several limitations. Firstly, this is a small cohort study of patients admitted with COVID-19 characterised by acute hypoxic respiratory failure and needing respiratory support may not be representative of all hospitalised COVID-19 patients. Secondly, the laboratory testing for D-Dimer was not consistently measured for all patients and the missing values may have introduced bias. Moreover, there was an upper limit cutoff for D-Dimer values of 5000 mg/l and as a result, we were unable to present the absolute laboratory values for levels beyond > 5000 mg/l. Finally, we did not perform additional renal specific urine biological investigations to characterize the AKI which limits our ability to evaluate the mechanism of renal injury. Whilst we recognise these limitations, we were still able to identify factors associated with the development of AKI and outcome of AKI recovery. Reassuringly, all but one patient who recovered from their acute illness have also completely recovered from their acute kidney injury.

## Conclusions

Acute kidney injury is a common feature in critically ill patients with COVID-19 pneumonia presenting with acute hypoxic respiratory failure. It is more common in patients with immunosuppression, hypertension and diabetes. The development of AKI is associated with increased severity of illness, prolonged duration of hospitalisation and increased mortality. Reassuringly, all surviving patients recovered from their acute kidney injury over time prior to their hospital discharge.

## Supplementary Information

**Additional file 1: Table S1.** Lymphocyte count 10^9^/l over the 7 days (Data presented in median (IQR)). **Table S2.** D-Dimer (mg/l) over the 7 days.

**Additional file 2.** The standard anticogulation protocol used in all ICU patients.

## Data Availability

The datasets used and/or analysed during the current study available from the corresponding author on reasonable request.

## Abbreviations

ACE: Angiotensin converting enzyme
AHRF: Acute hypoxic respiratory failure
AKI: Acute kidney injury
APACHE II: Acute Physiology and Chronic Health Evaluation II
ARB: Angiotensin receptor blocker
COVID-19: Coronavirus disease 2019
CVVHDF: Continuous veno-venous hemodiafiltration
HIV: Human immunodeficiency virus
ICU: Intensive care unit
KDIGO: Kidney Disease Improving Global Outcome
RRT: Renal replacement therapy
SARS-CoV-2: Severe acute respiratory syndrome coronavirus 2
SOFA: Sequential Organ Failure Assessment
WHO: World Health Organisation
