# Supplementary material for: Clinical characteristics and outcome of critically ill COVID-19 patients with acute kidney injury: a single centre cohort study
Source: BMC Nephrol. 2021 Mar 15;22:92. doi: 10.1186/s12882-021-02296-z (PMC7957445; doi:10.1186/s12882-021-02296-z)
Supplement: Supplementary file 1 — Additional file 1: Table S1. Lymphocyte count 109/l over the 7 days (Data presented in median (IQR)). Table S2. D-Dimer (mg/l) over the 7 days. [file 12882_2021_2296_MOESM1_ESM.docx]

Appendix 1

Table S1: Lymphocyte count 10^9^/l over the 7 days (Data presented in median (IQR)).

|  | Day 1 | Day 2 | Day 3 | Day 4 | Day 5 | Day 6 | Day 7 |
| --- | --- | --- | --- | --- | --- | --- | --- |
| AKI | 0.7 (0.45) | 0.8 (0.55) | 0.75 (0.75) | 0.7  (0.6) | 0.7 (0.85) | 0.7 (0.52) | 0.8 (0.45) |
| No AKI | 1.0  (0.5) | 0.95 (0.55) | 1.0  (0.6) | 1.0 (0.62) | 1.1 (0.58) | 1.2  (0.8) | 1.4  (0.7) |

Table S2: D-Dimer (mg/l) over the 7 days

|  | Day 1 | Day 2 | Day 3 | Day 4 | Day 5 | Day 6 | Day 7 |
| --- | --- | --- | --- | --- | --- | --- | --- |
| AKI | 1381  (2867) | 3035  (3716) | 1040  (2836) | 878  (1689) | 1518  (2264) | 1339  2541) | 1717  (2362) |
| No AKI | 477  (252) | 562  (396) | 534  (704) | 655  (648) | 628  (547) | 724  (651) | 743  (1155) |
