# Supplementary material for: Clinical characteristics and outcome of critically ill COVID-19 patients with acute kidney injury: a single centre cohort study
Source: BMC Nephrol. 2021 Mar 15;22:92. doi: 10.1186/s12882-021-02296-z (PMC7957445; doi:10.1186/s12882-021-02296-z)
Supplement: Supplementary file 2 — Additional file 2. The standard anticogulation protocol used in all ICU patients. [file 12882_2021_2296_MOESM2_ESM.pdf]

Thromboprophylaxis of patients on HDU/ICU with COVID-19 at University Hospital Southampton

Key Points:

1. **Hospitalised COVID-19 patients are at high VTE risk**
2. **A high incidence of VTE has been reported in COVID-19 patients on ICU despite standard dose thromboprophylaxis**
3. **A high D-dimer ± disseminated intravascular coagulation are associated with poor prognosis**
4. **There is evidence LMWH prophylaxis is associated with reduced mortality in severe COVID-19**
5. **Only give platelet transfusions if platelets <20 with minor/no bleeding, <50 and moderate bleeding or <75 and major bleeding**
6. **Only give FFP if INR >1.5 and moderate-severe bleeding**
7. **Only give cryoprecipitate if fibrinogen is <2 and moderate-severe bleeding**

All patients admitted with COVID-19 should have a UHS VTE risk assessment on admission

↓

Prophylactic enoxaparin* if no contraindications and platelets ≥ 50

↓

patient admitted to HDU/ICU?

No → Repeat VTE assessment within 24 hours of admission and when there is a clinical change

Yes ↓

1.Check FBC, clotting screen and d-dimer daily
2.Use prophylactic heparin** for first 24 hours on ICU while bleeding risk and renal function assessed
3.After 24 hours switch to enoxaparin prophylaxis* if no contraindications
4.Give VTE prophylaxis if platelets >30 , even if coagulopathy (raised INR/APTR ± low fibrinogen), unless moderate-severe bleeding

↓ discharge from ICU

Standard UHS VTE assessment and thromboprophylaxis, unless ongoing need for high dose enoxaparin prophylaxis

* If patient already on treatment dose anticoagulation consider switching to treatment dose enoxaparin

**See enoxaparin and heparin dosing guide below

Further information:
University Hospital Southampton Thromboprophylaxis and Risk Assessment Guideline: Appendix C Risk Assessment
BSH Interim Guidance:
https://thrombosisuk.org/downloads/T&H%20and%20COVID.pdf
ISTH Interim Guidance:
https://onlinelibrary.wiley.com/doi/epdf/10.1111/jth.14810
ISTH Academy:
https://academy.isth.org/isth/#!*menu=8*browseby=8*sortby=2*label=1979

April 2020: version 1
Authors: Dr Sara Boyce (consultant haematologist)
Dr Kathleen Nolan (critical care consultant)
Dr Rashid Kazmi (consultant haematologist)

Thromboprophylaxis of patients on HDU/ICU with COVID-19 at University Hospital Southampton

<table>
<tr><th>Weight</th><th>Enoxaparin dose for VTE prophylaxis [1,2,3]</th><th>Heparin dose for VTE prophylaxis [1,2,3]</th><th>Anticoagulation prophylaxis with haemofiltration</th></tr>
<tr><td><50 kg</td><td>Enoxaparin 40mg od</td><td>Heparin 5000 units bd</td><td rowspan="4">Intravenous heparin (APTR 1.5-2.5)

If unable to obtain target APTR despite increasing doses of heparin or patient clots despite target APTR send anti Xa levels (target 0.3-0.7)[4]</td></tr>
<tr><td><100 kg</td><td>Enoxaparin 40mg bd</td><td>Heparin 5000 units tds</td></tr>
<tr><td>100-150 kg</td><td>Enoxaparin 60mg bd</td><td>Heparin 5000 units tds</td></tr>
<tr><td>>150 kg</td><td>Enoxaparin 80mg bd</td><td>Heparin 7500 units tds</td></tr>
</table>

1.Enoxaparin doses should be reduced by 50% if creatinine clearance 15-30. If creatinine clearance <15 give s/c unfractionated heparin prophylaxis

2.Standard enoxaparin/heparin prophylactic doses (see UHS VTE risk assessment policy) should be given if platelet count 30-50, enoxaparin/heparin should be omitted if platelet count <30

3.All patients should have their anti Xa levels monitored to ensure adequate anticoagulation (see below)

4. If patient not achieving target anti Xa levels, or clotting despite therapeutic enoxaparin/heparin, check antithrombin, factor VIII and VWF antigen and discuss with a senior haematologist

All patients should be assessed for need for mechanical thromboprophylaxis using UHS VTE risk assessment

Thromboprophylaxis of patients on HDU/ICU with COVID-19 at University Hospital Southampton

Anti Xa monitoring of enoxaparin

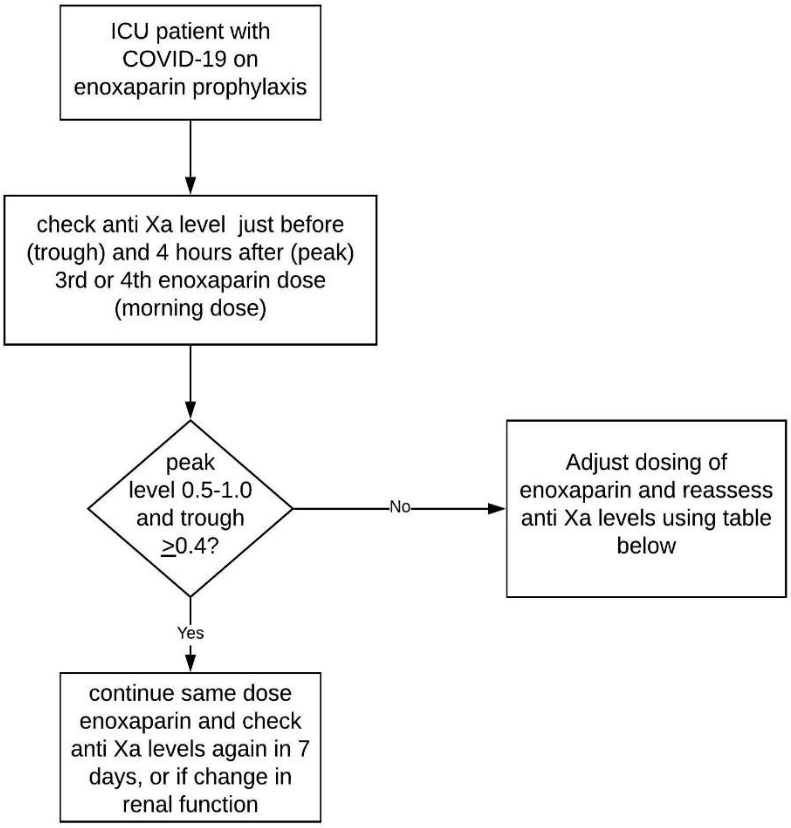

**Peak anti Xa level**	**Delay next dose**	**Enoxaparin dose change**	**Recheck peak (4 hour) anti Xa levels**
<0.35	no	Increase 25%	After next morning dose
0.35-0.49	no	Increase 10%	After next morning dose
0.5-1	no	No change	7 days, or if change in renal function
1.1-1.5	no	Decrease 20%	After next morning dose
1.6-2.0	3 hours	Decrease 30%	After next morning dose
>2	Until anti Xa <0.6	Decrease by 40%	When next dose due, then 12 hourly until anti Xa <0.6

Thromboprophylaxis of patients on HDU/ICU with COVID-19 at University Hospital Southampton

If trough level anti Xa <0.4 and peak level in/above target range discuss with haematologist (clotting phone 007585880224)

Anti Xa monitoring of subcutaneous heparin prophylaxis

- Anti Xa levels should be checked 6 hours after the first morning heparin dose, if the patient is not being converted to enoxaparin or IV heparin within the next 24 hours
- Aim for a target of anti Xa 0.2-0.5
- If level <0.25 increase heparin dose by 25%
- If level >0.5 reduce heparin dose by 25%
- After dose changes recheck the anti Xa level 6 hours after the next morning dose
